# Evaluation of Pain Regression in Patients with Temporomandibular Dysfunction Treated by Intra-Articular Platelet-Rich Plasma Injections: A Preliminary Report

**DOI:** 10.1155/2014/132369

**Published:** 2014-08-03

**Authors:** M. Pihut, M. Szuta, E. Ferendiuk, D. Zeńczak-Więckiewicz

**Affiliations:** ^1^Department of Dental Prosthetics, Consulting Room of Temporomandibular Joint Dysfunctions, Jagiellonian University, Medical College, 4 Montelupich Street, 31-155 Krakow, Poland; ^2^Department of Cranio-Maxillofacial, Oncological and Reconstructive Surgery, Jagiellonian University, Medical College, 1 Zlotej Jesieni Street, 31-826 Krakow, Poland; ^3^Department of Dental Surgery, Wroclaw Medical University, 26 Krakowska Street, 50-425 Wroclaw, Poland

## Abstract

*Objective.* The objective of this study was to evaluate the regression of temporomandibular pain as a result of intra-articular injections of platelet-rich plasma (PRP) to patients with temporomandibular joint dysfunction previously subjected to prosthetic treatment. *Materials and Methods.* The baseline study material consisted of 10 patients, both males and females, aged 28 to 53 years, previously treated due to painful temporomandibular joint dysfunction using occlusal splints. All patients were carried out to a specialist functional assessment of the dysfunction using the Polish version of the RDC/TMD questionnaire axis I and II. Intra-articular injections were preceded by a preparation of PRP. The injection sites were determined by the method used during arthroscopic surgical procedures. Following aspiration, 0.5 mL of plasma was injected into each temporomandibular joint. *Results.* The comparison of the intensity of pain during all examinations suggests a beneficial effect of the procedure being performed as the mean VAS score was 6.5 at examination I, 2.8 at examination II, and 0.6 at examination III. *Conclusion.* Application of the intra-articular injections of platelet-rich plasma into the temporomandibular joints has a positive impact on the reduction of the intensity of pain experienced by patients treated for temporomandibular joint dysfunction.

## 1. Introduction

A number of conservative methods are used in the treatment of temporomandibular joint dysfunction pain syndrome, including occlusal splints of various designs, supportive physical therapy procedures, rehabilitation involving muscular training, and specialist psychological support [1–10].

Intra-articular administration of medications is an established method of treatment, particularly in orthopedic and rheumatic disorders associated with pain, effusion, inflammation of cartilage, and bone and joint capsules as well as fibrous adhesions. Currently, agents used for intra-articular injection within the temporomandibular joint regions include hyaluronic acid and steroids [11, 12].

Although autogenous platelet-rich plasma (PRP) concentrates were developed in the 1970s, the medical use of these materials was facilitated only by subsequent technological advances, particularly by progress in medical instrumentation. In 1997, Whitman et al. were the first to present a method for preparation and use of platelet-rich plasma to accelerate healing processes. Centrifugation of full autogenous blood yields a platelet concentrate with platelet concentration of ca. 1 million cells per cubic millimeter of plasma. The concentrate is produced in the process of collecting peripheral vein blood (most commonly from the ulnar vein) directly from patients, with blood being centrifuged during the procedure. To date, platelets were demonstrated to contain more than 30 growth factors, the most important of them being the platelet derived growth factor (PDGF), transforming growth factor (TGF), epidermal growth factor (EGF), insulin-like growth factor (IGF), and vasoendothelial growth factor (VEGF) [13–21].

Platelets are anuclear, flat disc-shaped cells formed in bone marrow; about 30% of all platelets are found in the spleen. The life span of platelet cells ranges from 8 to 12 days; cells are eliminated by the mononuclear phagocyte system. When in the interphase, platelets have no thrombogenic potential; thrombin is required for their activation which involves a change in the shape and the release of granules. The main function of the platelets is the control of hemostasis. Platelets form hemostatic plugs at injury sites, participating in blood coagulation processes. Wound healing starts with platelet thrombus formation, platelet degranulation, and the release of growth factors. The next stage involves fibroplasia and angiogenesis followed by the synthesis of structural connective tissue elements. This leads to increased resistance to wound disruption and then to scar remodeling. New collagen is generated owing to chemotactic and mitogenic effects of FGF on fibroblasts, stimulating proliferation and differentiation of keratinocytes thanks to the presence of highly concentrated growth factors [12, 16, 18].

Platelet-derived growth factors contained in the concentrate are peptides that stimulate proliferation, differentiation, and migration of cells. These properties determine the usefulness of growth factors in the healing process. Most growth factors present within the platelet-rich plasma have mitogenic properties leading to an increase in the repair cell counts. Growth factors derived from centrifuged blood were used for the first time by Knighton in patients with chronic skin ulcerations [18].

Platelet-rich plasma contains proteins that are responsible for cell adhesion, namely, fibrin, fibronectin, and vitronectin. It stimulates tissue regeneration processes by stimulating fibroblasts to produce structural proteins for use in formation of new collagen and elastin, support of remodeling and angiogenesis (formation of new vessels), and activation of mesenchymal stem cells. PRP is widely used in surgical procedures, in the treatment of burns and extensive, difficult-healing wounds, orthopedic ligament injuries, and connective tissue injuries. An additional advantage of this method is the safety of the material and a zero possibility of hypersensitivity to injected plasma. It should be kept in mind that blood and plasma may be carriers of pathogens such as HIV, CMV, EBV, or HCV, and therefore precautions should be used consisting of appropriate staff apparel and patient behavior [13–18, 21, 22]. (Consent of the Bioethics Committee Number KBET/125/L/2013.)

The objective of this study was to evaluate the regression of temporomandibular pain as a result of intra-articular injections of platelet-rich plasma to patients with temporomandibular joint dysfunction previously subjected to prosthetic treatment.

## 2. Materials and Methods

The baseline study material consisted of 10 patients, both males and females, aged 28 to 53 years, previously treated using occlusal splints, who reported at the Consulting Room of Temporomandibular Joint Dysfunctions of the Department of Dental Prosthetics of the Jagiellonian University Medical College in Krakow between 01.09.2013 and 20.05.2014 due to painful dysfunction of the stomatognathic system.

All patients were carried out to a specialist functional assessment of the masticatory organ using the Polish version of the RDC/TMD questionnaire axis I and II (examination I). The following parameters were analyzed in detail for the purposes of this study: changes in the intensity of pain as reported by the patients with regard to temporomandibular joints and stomatognathic system muscles, pain intensity characteristics, level of impairment, and grade of chronic pain [23, 24].

The assessment involved also a subjective assessment of pain within the stomatognathic system muscles and temporomandibular joints, evaluation of the range and symmetry of mandible motion, acoustic symptoms within the temporomandibular joints, and the impact of the masticatory organ health on overall well-being [1, 3, 23].

The inclusion criteria included temporomandibular joint dysfunction pain manifested as lack of coordination in operation of articular heads and disc (disc translocation with or without disc blockade/crackling sounds/) and dysfunctions presenting with excessive masticatory muscle tone as the dominant problem and lack of benefits from previous treatment methods. All subjects reported that the pain had sustained for at least 3 months and that they continued the prosthetic treatment.

The exclusion criteria included poor overall health, unwillingness to participate, numerous dental defects, diagnosis of a connective tissue disease, and dysfunctions classified as RDC/TMD groups IIIb and IIIc. Contraindications associated with the use of platelet-rich plasma (platelet function disorders, fibrinogen deficiency, and anticoagulation treatment) were also taken into account [1, 3, 5, 6, 11, 23, 24].

**Figure 1:**
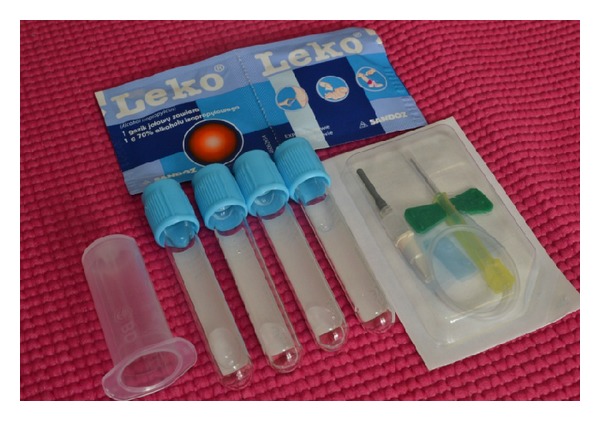
Disposable closed set for blood collection.

Intra-articular injections of platelet-rich plasma were preceded by collection of peripheral blood from the ulnar vein of the patient in accordance witj the procedures in force using single-use, closed vacuum systems and glass tubes with sodium citrate as an anticoagulant (Figure 1). After mixing the collected blood exactly with the citrate using rotational movements, an even number of tubes were placed in a centrifuge rotor. Centrifugation parameters were set to 3,200 rpm, and the centrifugation time was 12 minutes. After separation of the erythrocytic mass and the platelet-poor and platelet-rich plasma layered directly above the erythrocytes, the platelet-rich plasma was aspirated with caution into a separate syringe. Thus prepared concentrate was ready for injection into the temporomandibular joint regions [18, 24–27].

**Figure 2:**
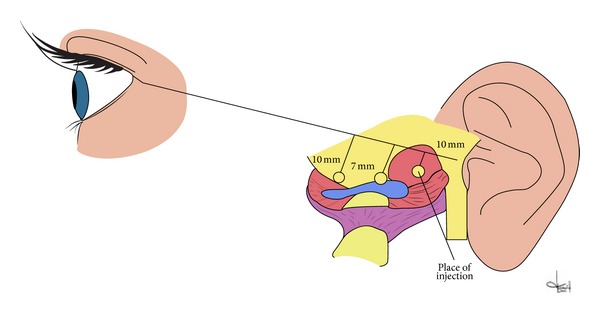
Scheme of place for intra-articular injection.

The injection sites were determined by the method used during arthroscopic surgical procedures within the temporomandibular joints. Patients were prepared for the procedure by a line being drawn on their skin between the earlobe and the outer eye corner. Three segments were marked at 10 mm intervals starting from the earlobe. The lengths of the lines were 3 mm (first line), 5 mm (second line), and 7 mm (third line). The platelet-rich plasma injection site was marked by the tip of the third line corresponding to the upper compartment and retrodiscal zone (Figure 2). The skin at the injection site was washed with a disinfectant to decontaminate the field. Injections were made in the determined point with the mandible abducted. Following aspiration, 0.5 mL of plasma was injected into each temporomandibular joint. The skin was disinfected once again after the injection. Patients were informed about the possibility of experiencing an unpleasant and transient sensation of fullness or compression in the joint regions. Clinical follow-up was performed 7 days (examination II) and 6 weeks (examination III) after the procedure [18, 28–30].

The following parameters were assessed in the specialist baseline examination and the follow-up examinations using the RDC questionnaire: the presence and intensity of spontaneous and palpation-induced pain within temporomandibular joints and masticatory muscles, the range and symmetry of mandible motion, abduction trajectory, and the presence of acoustic symptoms within temporomandibular joints during all mandible movements and accompanying symptoms such as squeaking sounds and tinnitus in ears. In one case, classified as RDC group IIb, active unblocking of the articular joint was first performed; the patient was subsequently qualified for platelet-rich plasma injection [8, 23].

The results were subjected to statistical analysis using the basic procedures such as determination of means, standard deviations, medians, and post hoc ANOVA Friedmana statistical significance test.

## 3. Results

The group of patients qualified for the study consisted of two males and eight females. The mean age was 37.6 years. According to the results of baseline examinations, the patients reported for prosthetic treatment for the following reasons: pain within one or both temporomandibular joints, spontaneous or occurring at mandible movements, chewing, tight jaw occlusion, or joint palpation. The pain was located in the joints or the masticatory muscles, radiating into the temporal and cranial region in 2 cases. Joint crackling sounds were heard upon mandible movements by 4 patients, while a reduced range of mandible abduction was observed in 1 patient. The following diagnoses were made on the basis of RDC/TMD questionnaire axis I survey: 1 case of group I a, 4 cases of group II a, case of group II b, and 4 cases of group III a (Table 1).

**Figure 3:**
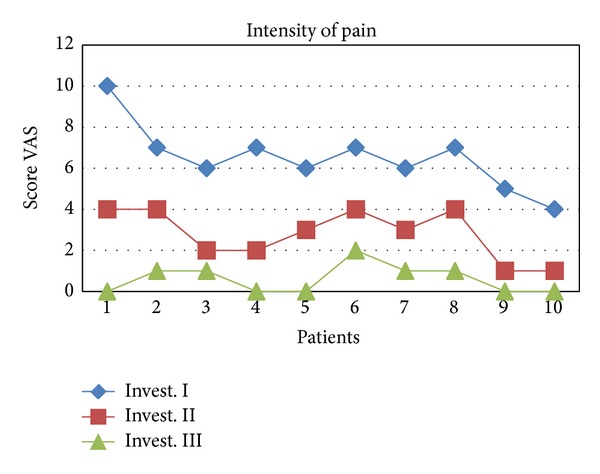
Values of pain scoring in the subsequent studies presented graphically.

**Figure 4:**
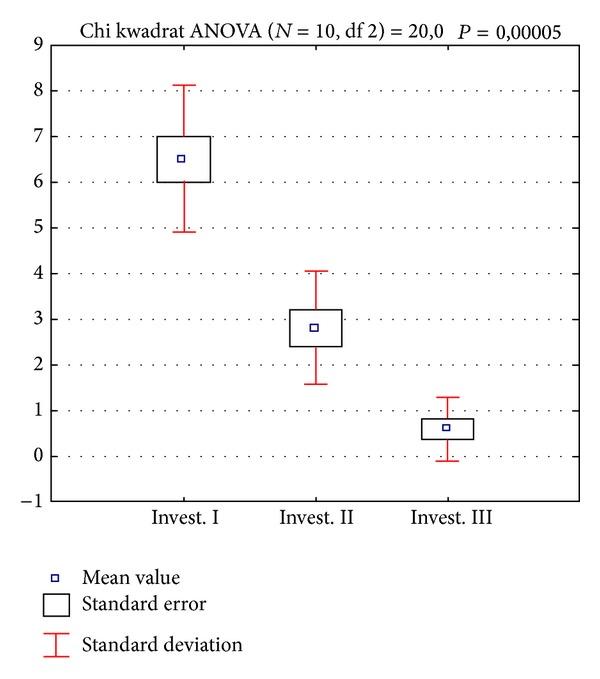
Average values of pain scoring in the subsequent studies in the statistical calculation, presented graphically.

The comparison of the intensity of temporomandibular joint and masticatory muscles pain at baseline and follow-up examinations suggests a beneficial effect of the procedure being performed as the mean VAS score was 6.5 at examination I, 2.8 at examination II, and 0.6 at examination III. The result of subsequent investigation differs in the statistically significant way (Tables 2(a) and 2(b), Figures 3 and 4). It should be mentioned that the question regarding the presence of pain within the last 6 months was answered positively by all patients and the minimum score marked at the VAS scale was 5. Acoustic symptoms observed within the temporomandibular joints in 4 cases (group II a) at examination I were experienced by only one patient in the final examination.

Table 3 presents the results of the RDC/TMD (axis II) evaluation of chronic pain in the study group, assessing the characteristics of pain intensity, impairment level, and chronic pain grade. The results showed grade I chronic pain in 2 subjects, grade II chronic pain in 4 subjects, grade III chronic pain in 1 subject, and grade IV chronic pain in 3 subjects.

## 4. Discussion

Chronic facial pain is a common cause of impairment in the everyday activities of patients and requires numerous therapeutic procedures being initiated by physicians of different dental specialties as well as by neurologists, laryngologists, maxillofacial surgeons, and psychiatrists. This suggests a need for interdisciplinary treatment due to the complex character of complaints. Temporomandibular joint pain is a special problem as the mandible motion upon chewing and talking constitutes a stimulus for a significant increase in the pain being experienced [1–10, 24, 29, 31–34].

Acoustic symptoms and tenderness on palpation (associated with hyperactivity) of mandible lifting muscles are an evidence of overloads located within the posterior disc ligament (strain, swelling, and corrugation). The area is poorly accessible for application of healing-promoting drugs and the physical therapy procedures applied in these cases are not always satisfactory.

Many researchers studying PRP treatments highlight the high efficacy of this method of management of musculoarticular disorders and its safety due to the use of autologous material as well as the low costs of treatment [12–22, 25–28, 30, 35–57].

Daif compared the efficacy of intra-articular injections within the upper temporomandibular joint compartment and additional injections in the articular capsule region points out the superiority of the latter method for the treatment of dysfunctions involving disc translocation with no disc blocking [58]. PRP is commonly used in dentistry procedures such as filling bone defects with trabecular bone chips mixed with platelet-rich plasma. The results of follow-up histomorphometric examinations performed 12 weeks after the procedure revealed higher bone density in patients having undergone the transplant of trabecular bone transplant combined with PRP [13].

Today, the use of platelet-rich plasma has been expanded no include numerous indications after orthopedic surgeries to treat sports-related injuries. PRP is widely used for tissue healing in many anatomical regions as well as in the treatment of pathological lesions of bone, cartilage, ligament, and muscle tissues. The popularity of this novel treatment method triggered a rapid increase in research. However, differences in application techniques and PRP compositions make comparisons of efficacy results difficult. Potential complications following the procedures involving PRP administration are mild and therefore this method of treatment appears to be safe. It brings various potentially positive effects for the damaged musculoskeletal system tissues. Platelet-rich plasma injection is used with increasing frequency in reconstructive orthopedic procedures (tennis elbow, knee injuries, healing of meniscus, cruciate ligaments, and Achilles tendons), muscle injuries, dental surgeries, and implantations [12–22, 27, 30, 35–40, 45–51]. As highlighted by Middleton et al., PRP injections promote optimization of healing environment and facilitate earlier functional rehabilitation of joints [42]. Studies conducted by Paoloni et al. and Pelletier et al. demonstrated high efficacy of PRP injections in the anterior cruciate ligament region in the knee and food injuries [46, 47]. Filardo et al. reported the use of platelet-rich plasma in the treatment of intra-articular cartilage injuries and knee inflammations; the treatment was reported to cause rapid reduction of pain and quick recovery of functional capability [27]. Studies conducted by Sundman et al. demonstrated significant anti-inflammatory properties of plasma [50]. Proper healing is also influenced by appropriate preparation of platelet-rich plasma, starting from proper collection of peripheral blood and optimum centrifugation parameters and ending at the administration procedure itself [12–22, 25–28, 30, 35–58].

A separate opinion suggesting the lack of confirmed benefits of PRP on musculoarticular tissue healing has been presented by Willits et al., who claim that the review of PubMed and Medline data performed in April 2013 does not warrant any positive opinions on the clinical use of this method [59].

The results of our preliminary research suggest beneficial effects of platelet-rich plasma in intra-articular injections as a supplement to the basic prosthetic treatment of temporomandibular joint dysfunction. The reduction in pain and gradual restoration of functional capabilities of the stomatognathic system as the result of the treatment of interest raises hopes that future studies would lead to establishment of appropriate management algorithms.

## 5. Conclusion

Conclusions are as follows.

1. Additional intra-articular injections of platelet-rich plasma into the temporomandibular joints have a positive impact on the reduction in the intensity of pain experienced by patients treated for temporomandibular joint dysfunction.
2. Determination of appropriate treatment algorithm making use of intra-articular injection in patients with temporomandibular joint dysfunction pain requires further studies and longer follow-up of the study group.

## Figures and Tables

**Table 1:** Categories of clinical form according to the RDC/TMD.

Patient	1	2	3	4	5	6	7	8	9	10

Clinical form	IIIa	IIb	IIa	IIIa	IIa	IIIa	IIa	IIIa	IIa	Ia

**Table:** (a)

Investigation	Patient									
	1	2	3	4	5	6	7	8	9	10
Invest. I	10	7	6	7	6	7	6	7	5	4
Invest. II	4	4	2	2	3	4	3	4	1	1
Invest. III	0	1	1	0	0	2	1	1	0	0

**Table:** (b)

ANOVA Friedmana Chi kwad. ANOVA (*N* = 10, df 2) = 20,0 *P* = 0,00005 0		
	Mean value	Standard deviation

Invest. I	6.50	1.58
Invest. II	2.80	1.23
Invest. III	0.60	0.70

**Table 3:** The results of scoring protocol for grade chronic pain.

Patient	Characteristic pain intensity (CPI)			Disability points	CPGC [grade]
	I Invest.	II Invest.	III Invest.		
1	100	80	66	5	IV
2	90	73	63	6	IV
3	43	33	30	0	I
4	63	46	40	2	II
5	63	53	43	1	II
6	70	60	53	3	III
7	40	26	23	0	I
8	70	60	50	5	IV
9	66	53	46	3	II
10	60	46	43	2	II

CPGC: chronic pain grade classification.
